# Supplementary material for: Bandgap Engineering On Demand in GaAsN Nanowires by Post‐Growth Hydrogen Implantation
Source: Small. 2025 Dec 17;22(7):e06091. doi: 10.1002/smll.202506091 (PMC12862452; doi:10.1002/smll.202506091)
Supplement: Supplementary file 1 — Supporting Information [file SMLL-22-e06091-s001.pdf]

NANO · MICRO
small

Supporting Information

for *Small*, DOI 10.1002/smll.202506091

Bandgap Engineering On Demand in GaAsN Nanowires by Post-Growth Hydrogen Implantation

*Nadine Denis, Akant Sagar Sharma, Elena Blundo, Francesca Santangeli, Paolo De Vincenzi, Riccardo Pallucchi, Mitsuki Yukimune, Alexander Vogel, Ilaria Zardo, Antonio Polimeni, Fumitaro Ishikawa and Marta De Luca**

Supplementary Information: Bandgap engineering on demand in GaAs/GaAsN nanowires by post-growth hydrogen implantation

*Nadine Denis Akant Sagar Sharma Elena Blundo Francesca Santangeli Paolo De Vincenzi Riccardo Pallucchi Mitsuki Yukimune Alexander Vogel Ilaria Zardo Antonio Polimeni Fumitaro Ishikawa Marta De Luca**

Nadine Denis, Alexander Vogel, Ilaria Zardo
Department of Physics, University of Basel, Basel, Switzerland
Dr. Akant Sharma, Dr. Elena Blundo, Francesca Santangeli, Paolo De Vincenzi, Riccardo Pallucchi, Prof. Antonio Polimeni, Prof. Marta De Luca
Dipartimento di Fisica, Sapienza Università di Roma, Rome, Italy
marta.deluca@uniroma1.it
Mitsuki Yukimune, Prof. Fumitaro Ishikawa
Graduate School of Science and Engineering, Ehime University, Ehime, Japan
Prof. Fumitaro Ishikawa
Research Center for Integrated Quantum Electronics, Hokkaido University, Sapporo, Japan

1 Band Anti-Crossing model for the nanowire samples in this study

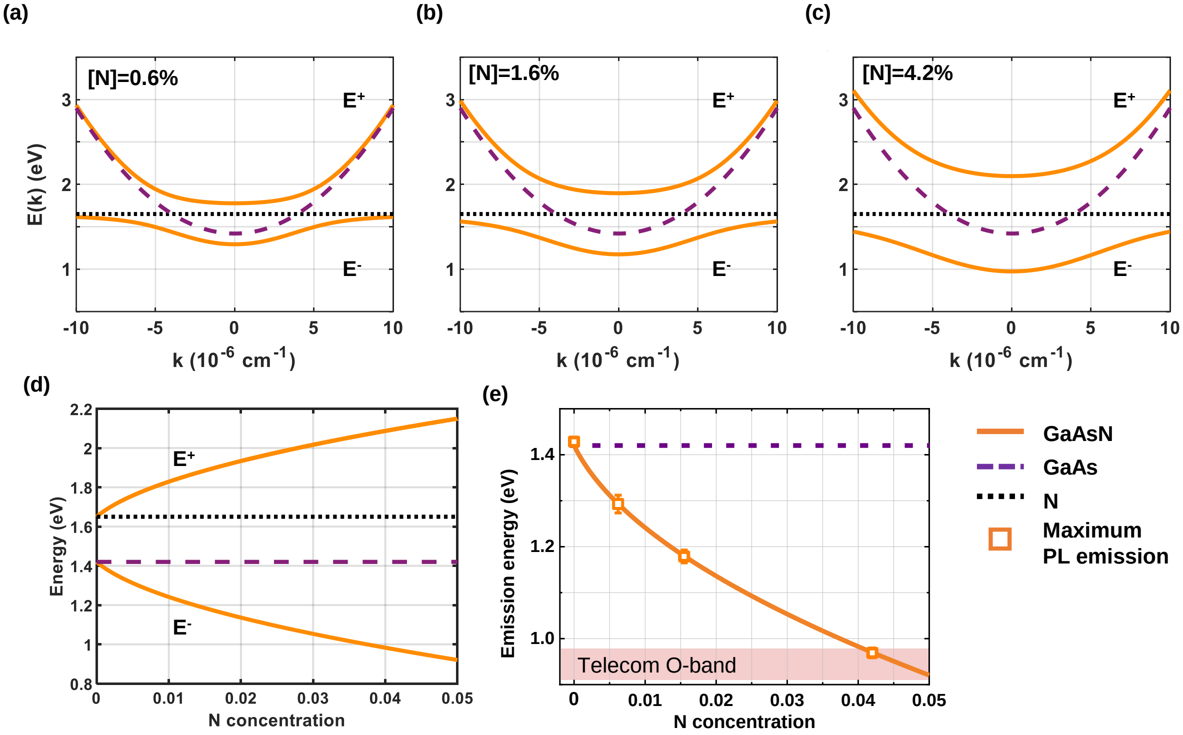

Figure 1: Bandgap energy estimation around the Γ-point using the bandgap anti-crossing model for dilute GaAsN in a quadratic approximation. (a-c) show the degenerate GaAs conduction band energy as a dashed line, the energy of isolated N-atoms as a dotted line and the upper and lower conduction band of GaAsN marked as E^+ and E^- with solid lines calculated for the different N concentrations of 0.6%, 1.6% and 4.2% respectively. (d) shows the minimum of the respective energy band as a function of N concentration and (e) shows the conduction band minimum together with the maximum of the PL emission from the nanowire samples with different N concentration with the telecom O-band marked as a shaded area. The calculation was done for $T = 295$K with using for the interaction potential $V = 2.7$eV, for the electron mass $m^* = 0.0635 m_e$, for the conduction band energy of GaAs $E^C = 1.42$eV and for the energy of the N-atoms $E^N = 1.65$eV[1, 2, 3].

In contrast to other ternary III-V semiconductor alloys, the bandgap of GaAsN does not follow Vegard's law, which is based on the virtual crystal approximation and assumes that the average lattice potential can be weighted

according to the concentration of the constituents. In particular, the bandgap energy of GaAs decreases when the N concentration is increased, while according to Vegard's law it would move towards the higher energy bandgap of GaN. The significant decrease of the GaAsN bandgap for dilute N concentrations is linked to a bowing of the GaAsN conduction band, which has been well described by the band anti-crossing (BAC) model [4]. The energy level of the isolated N atom in the GaAs lattice is about 250 meV above the minimum of the degenerate conduction band of GaAs. Within the BAC, the narrow N states are considered to be in resonance with the conduction band and act as a perturbation potential. The interaction of these two types of states leads to the following eigenvalue problem:

$$\begin{vmatrix} E - E^C & V^{CN} \\ V^{CN} & E - E^N \end{vmatrix} = 0, \tag{1}$$

where E^C and E^N are the energies of the conduction band edge and the N-level with respect to the top of the valence band. V^{CN} is the matrix element for the interaction and mixing of these states and depends on the N concentration x. It can be defined as $V^{CN} = V^2 \cdot x$. The solution of the eigenvalue problem shows a splitting of the previously degenerate conduction band and results in the following dispersion relation for an upper and lower band:

$$E^{\pm}(k) = \frac{E^N + E^C(k) \pm \sqrt{(E^N - E^C(k))^2 + 4V^2 \cdot x}}{2}. \tag{2}$$

The model correctly predicts the splitting of the conduction band into two subbands and the downward shift of the lower E_{-} branch, which leads to the reduction of the fundamental bandgap observed in PL measurements. The results of the BAC behavior of the GaAs conduction band are shown in **Figure** 1. (a-c) show the dispersion relation around the conduction band minimum for the N concentrations of 0.6%, 1.6% and 4.2%, which correspond to the N concentrations in the GaAsN shell of the nanowire samples investigated in this work. As the N concentration increases, the anti-crossing behavior of the bands is stronger and the conduction band minimum shifts to a lower energy. The dependence of the minimum of the upper and lower conduction band on the N concentration is shown in (d). (e) partially reproduces a graph shown in the main article, which shows the maximum of the PL emission peak at RT superimposed on the emission energy calculated as a function of N concentration according to the BAC.

2 Statistics on Bandgap Emission: Pre- and Post-Hydrogen Implantation

Figure 2 shows the statistics to understand the distribution of the GaAsN bandgap energy across different points on various single nanowires and its changing after hydrogenation. The data from different nanowire samples with N concentrations of 0%, 0.6%, 1.6% and 4.2% are shown in columns form (a)-(d). The top row shows data from different untreated nanowires and the bottom row shows data taken after H implantation with a dose of $d_h = 0.8 - 1.2 H_0$ with $H_0 = 10^{19}$ $\mathrm{H}^*/\mathrm{cm}^2$. Specifically, the plots show the energy at the maximum of the PL emission from room temperature (RT) μ-photoluminescence (PL) spectra. The purple dashed lines indicate the average emission energy and the black dashed lines indicate the standard deviation which gives an estimation of the variations between different points on different nanowires and is used for the error bars in Figure 1 (c) of the main article. The scale of the y-axis was chosen to span the same energy range for all plots to make the distribution of the data points from different samples comparable. In all N-containing samples, the bandgap energy of the untreated nanowires is distributed over a wider range compared to the hydrogenated nanowires. This is because small fluctuations in the N concentration have a big impact on the bandgap and are typical for such high GaAsN concentrations grown under non-equilibrium conditions in an MBE process. These fluctuations can be exacerbated by zincblende (ZB) and wurtzite (WZ) politypism, which may lead to different N incorporation into the GaAs lattice depending on the local crystal structure present in these nanowires. These local fluctuations of the potential induced by N change the bandgap energy locally, thus resulting in considerable point-to-point variations. During hydrogenation, H binds to the N atoms passivating their impact on the GaAsN bandgap and thereby reduces the linewidth broadening and bandgap energy variations caused by N concentration fluctuations. Moreover, even for a same N content, slightly different bandgap energies could arise from the different crystal structures. **Figure 3** shows the full width at half maximum (FWHM) of the PL band of the different

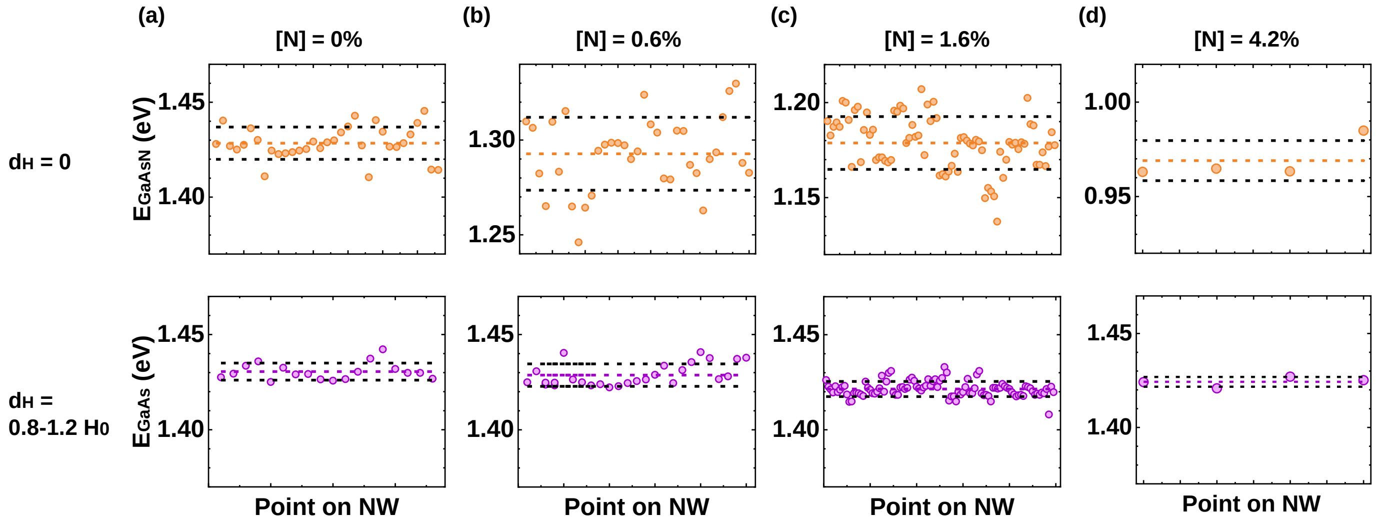

Figure 2: Distribution of the GaAsN bandgap emission in different points before and after H implantation. (a)-(d) show the peak energy of the RT PL spectra of the GaAsN shell before and after hydrogenation for the different N concentrations of 0%, 0.6%, 1.6% and 4.2%. The top row shows the measurements before hydrogenation and the bottom row the measurements after hydrogenation with a dose between $d_h = 0.8 - 1.2H_0$. Each point corresponds to a spectrum taken in a different point on several single nanowires. The horizontal purple dashed line is the mean value of the emission energy and the two black lines show the standard deviation used to estimate the error bar in Figure 1 of the main article. The standard deviation gives a measure of how much the bandgap energy varies in different points and different nanowires.

N concentration	Pristine (cts/s)	Hydrogenated (cts/s)
0	100	11400
0.6%	160	320
1.6%	710	21400
4.2%	1250	5130

Table 1: This table reports the maximum PL intensity that gives the normalization factors for the spectra shown in Figure 1 of the main paper in counts/s. The excitation power is $P = 10$ µW for N concentrations of 0, 0.6% and 1.6% and $P = 50$ µW for a N concentration of 4.2% due to a lower detector efficiency in this energy range.

samples from (a-d). The data of the pristine samples are shown in the top row and the data of the hydrogenated samples are shown in the bottom row. The FWHM is calculated throughout the whole paper as the linewidth of the emission band measured at half maximum. The same N-concentration fluctuations mentioned above lead to a larger FWHM for the N-containing nanowires. We observe a reduction by approximately a factor 2 for the hydrogenated samples, resulting in similar values as for the GaAs reference sample.
Table1 reports the normalization factors from Figure 1 of the main paper. A general increase in luminescence is observed after hydrogenation. Remarkably, we do not observe a significant quenching for increasing N concentration, which highlights the good crystal quality of this lattice-mismatched core-shell heterostucture in the nanowire geometry.

3 Effects of different hydrogenation conditions

Determining the correct H dose is crucial for an effective hydrogenation of GaAsN-based nanowires. If the dose is too low, not all N atoms are passivated; if the dose is too high, undesired defects may be created. Apart from the H dose, there are two main parameters affecting the hydrogenation process, namely the ion beam energy and the sample temperature during hydrogenation. The beam energy mainly affects the capability of H to penetrate into the nanowire and the creation of possible defects in the area close to the surface. The sample temperature influences the diffusion properties of H within the nanowire crystal lattice. Furthermore, certain H-complexes may be stable only at lower temperatures. In the following section, these three parameters are analyzed with further data on more nanowires and additional conditions to complement those presented in the main article. In general, we would like to emphasize the importance of maintaining the same beam energy and hydrogenation

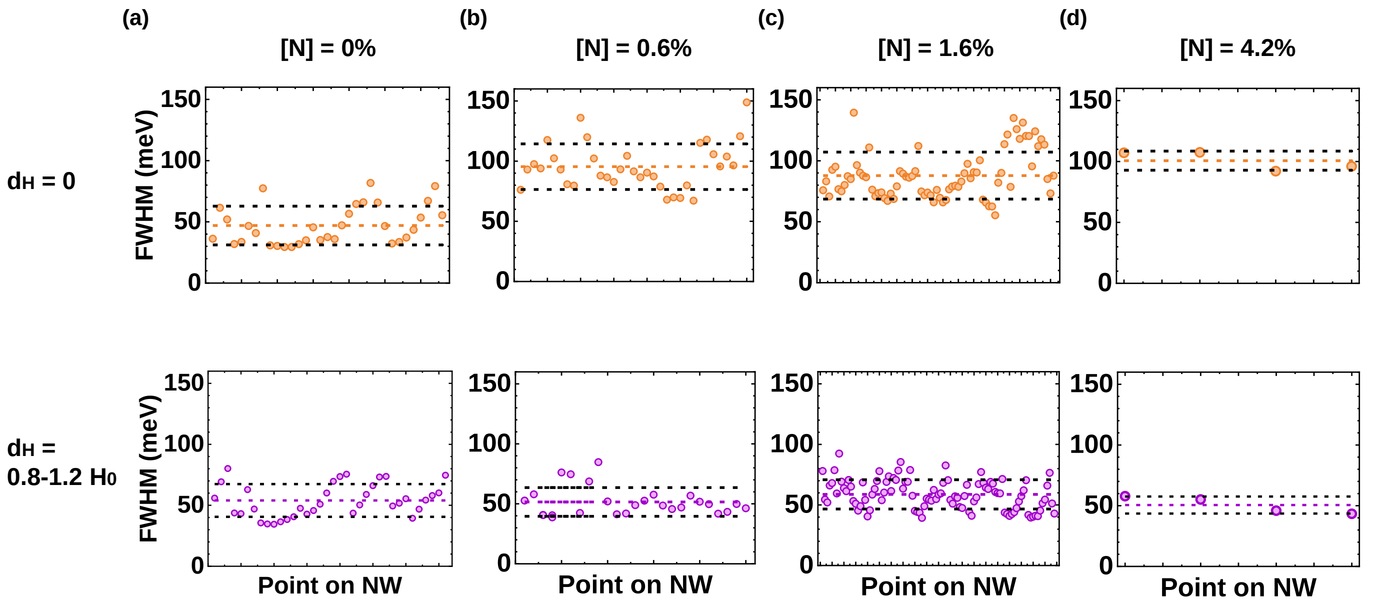

Figure 3: (Ex.: Distribution of the linewidth for the GaAsN bandgap emission in different points before and after H implantation.) (a)-(d) show the FWHM of the RT PL emission of the GaAsN shell for the different N concentrations of 0%, 0.6%, 1.6% and 4.2%. The top row shows the measurements before hydrogenation and the bottom row the measurements after hydrogenation with a dose $d_h = 0.8 - 1.2H_0$. Each point corresponds to the FWHM of the GaAsN band emission in a different point of several single nanowires. The purple line is the mean value of the FWHM and the black lines show the standard deviation used to estimate the error bar in Figure 1 of the main article.

temperature in order to find the perfect dose for a particular sample.

3.1 Increasing H-dose for other N-concentrations

In **Figure 4** we investigate the effects of increasing H-dose on the PL emission of samples with 0.6% N in (a-c) and of the GaAs reference nanowires in (d-f), complementing the analysis done on the samples with 1.6% N that is presented around Figure 2 in the main article.

0.6% N in the GaAsN shell: Figure 4 (a) shows μ-PL spectra measured at the same point of a nanowire after consecutive hydrogenations at a hydrogenation temperature of $T_H = 230°C$ with a beam energy of 120 eV. The pristine nanowire shows a strong emission from the low bandgap energy GaAsN shell, the maximum peak emission has 2000 counts/s when excited with a laser power of $P = 5\mu W$. The GaAs core emission is visible only as a tiny peak before hydrogenation. At a very low H dose of $0.01H_0$ no obvious shift of the GaAsN bandgap energy is visible. By increasing the H dose by an order of magnitude to $0.1H_0$, part of the N atoms in the GaAsN shell are being passivated, leading to more and more emission at the bandgap energy of GaAs. The spectrum with a dose of $1.3H_0$ shows the highest emission intensity at the maximum of the peak with 4490 counts/s compared to the maximum emission of the untreated nanowire of 2160 counts/s. At high H dose of $3.3H_0$ the nanowire is oversaturated with H, with all N centers already being passivated. The excess H leads to the emission of a new emission band between 1.1 and 1.3 eV which appears similarly in samples with other N concentrations and the pure GaAs reference samples. Comparing the ratio of the integrated PL emission intensity above and below 1.36 eV as a function of injected H in (b), we find a range for an optimal hydrogenation dose of $0.8 - 1.1H_0$. The data is from PL measurements on different points in several nanowires. We expect the optimal dose to vary depending on the thickness of the nanowire, which changes along the nanowire axis, and depending on the presence of local defects that might bind to H atoms during the hydrogenation. (c) shows the ratio of the integrated emission over the whole PL spectrum before and after hydrogenation of the single measurements in several points on various nanowires with the mean indicated as a black cross. The differences in intensity gain or loss between different points become apparent in this plot. On average, we observe a slight de-

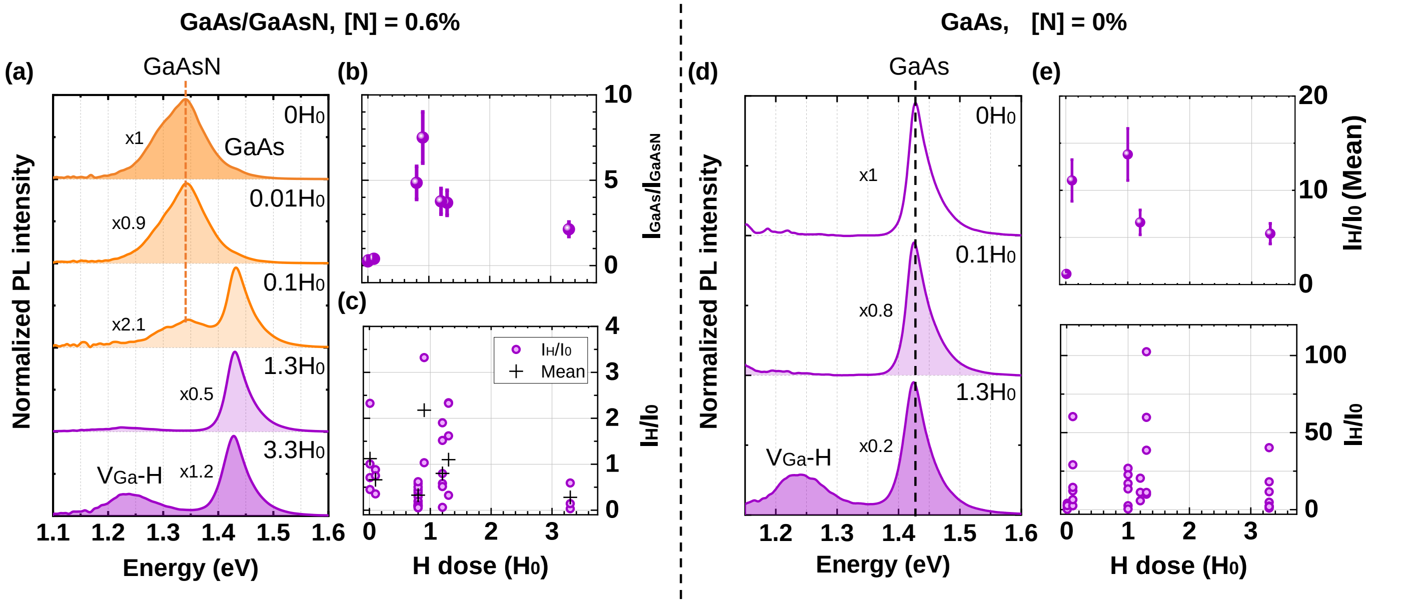

Figure 4: Effects of increasing H dose on the nanowire samples with 0.6% N in (a-c) and for the GaAs reference nanowires in (d-e). (a) and (d) show selected RT PL spectra for increasing H dose measured at the same location of each nanowire after successive hydrogenations. The spectra are normalized to the maximum PL intensity before hydrogenation. (b) shows the ratio of the integrated RT PL intensity of the GaAs-like emission divided by the integrated low-energy emission of the GaAsN or V_{Ga}-H peak. (c) shows the change in PL intensity as a ratio between the integrated intensity after and before hydrogenation; each point corresponds to a different measurement, the mean value is marked as a black cross. (e) shows the increase in RT PL intensity of the GaAs reference sample with increasing H dose, measured as the ratio of the integrated PL emission of the GaAs peak after and before H implantation. The graph above shows the average value calculated by excluding outliers more than 2 standard deviations away from the mean. The data points from the individual measurements in the lower graph show a very high increase in PL intensity in certain points that were not taken into account to understand a general trend.

crease in overall PL intensity for this low N concentration nanowire sample after hydrogenation. However, there are some individual points that show some increase in PL emission.

GaAs reference nanowires (0% N in the GaAsN-shell): Figure 4 (d) shows a point on a GaAs reference nanowire measured after consecutive hydrogenation. The sample is hydrogenated at a temperature of $T_H = 230°C$ with a beam energy of 120 eV. At a low H dose, the lineshape of the PL spectrum is not affected by the hydrogenation. At a high H dose above $1H_0$, however, a new emission band appears at energies between 1.1 - 1.3 eV, similar to the one observed for high dose in N containing samples. This proves, that the appearance of this band for high H-dose is not linked to the presence of N in the lattice. We identify it as a H-donor to Ga-vacancy (V_{Ga}-H) transition that is activated when the H-dose is high enough, for the H atoms to get trapped by the Ga vacancy. To understand the influence of the H-dose on the PL intensity, the ratio of the integrated PL emission of the GaAs peak measured at the same point before and after hydrogenation is compared for different H-dose shown in (e). The mean value is shown on top and the individual data points in the bottom row. We observe a H induced increase in PL intensity up to a dose of about $1H_0$ by a factor of 13 on average. The plot of the individual measurements shows a wide spread between the measurements at different points on nanowires hydrogenated under the same conditions. This is related to a stronger increase in PL intensity near non-radiative emission centers that are deactivated by H. In order to calculate a meaningful trend for the dependence on the H-dose, the average is calculated by removing the outliers with a distance of two standard deviations from the mean value. This procedure is used for all samples with different N-concentrations. Further discussions are found in the main article.

3.2 Different Beam energy

The variation of the energy of the H atoms in the ion beam affects how deeply and at what speed the H atoms penetrate into the GaAs shell before they begin to diffuse to other locations in the nanowire lattice. It is known that proton implantation may cause defects in the near-surface region. The higher the beam energy, the more defects are created. However, if the energy is too low, the H atoms could remain on the surface. Another factor to

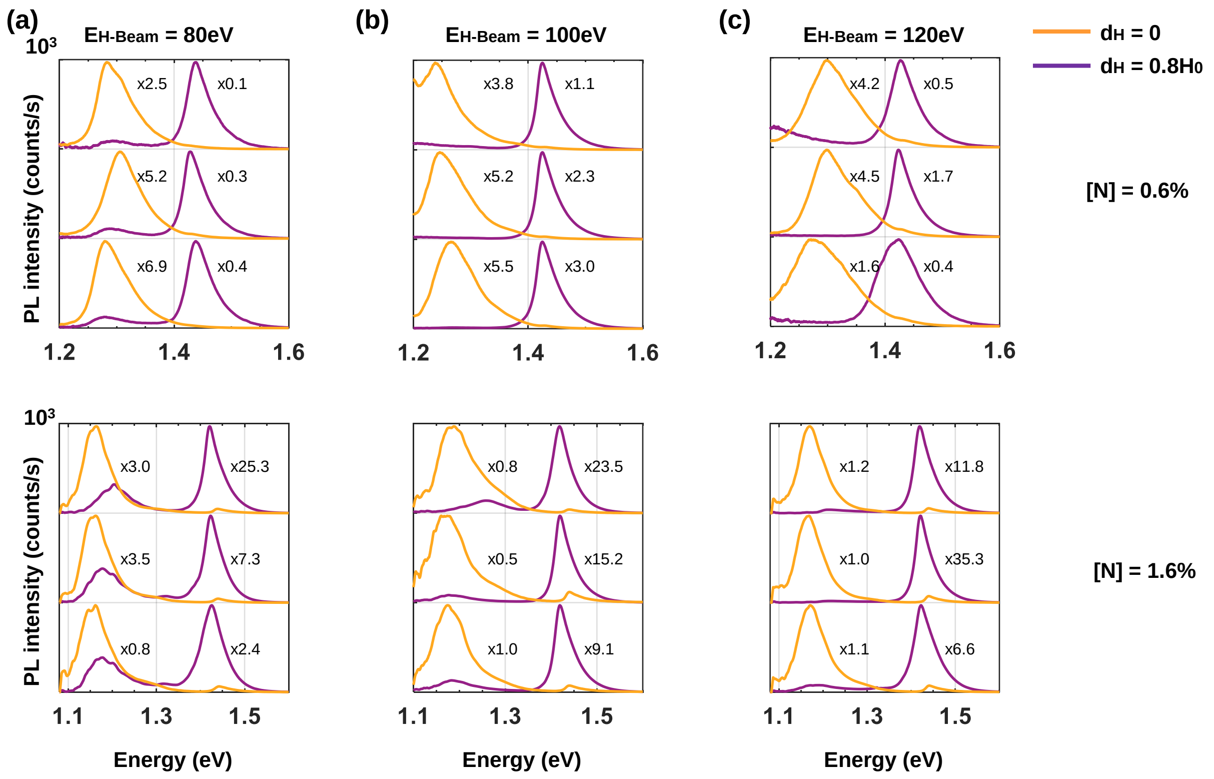

Figure 5: Effects of hydrogenation with beam energies of 80, 100 and 120 eV on the PL emission. The H dose of $d_H = 0.8H_0$ and hydrogenation temperature of $T_H = 230°C$ are kept the same. (a-c) show three RT PL spectra for each hydrogenation energy at different points measured before and after hydrogenation for nanowires with 0.6% N and (d-f) for 1.6% N respectively. Each spectrum is normalized to the maximum indicated by the factor next to it, the intensity trends are discussed thoroughly in Figure 5.

consider is the fact that higher beam energies allow higher proton currents in the Kaufmann source, which significantly shortens the hydrogenation time. The hydrogenation time for the samples was 24, 15 and 5 hours to achieve the same H dose with beam energies of 80, 100 and 120 eV, respectively. In a study on changes in the intensity of the PL emission from nanowires after annealing for a different time, we have observed a decrease of the PL intensity after 1h but only little change when annealed for a longer time. We conclude that the annealing at $T_H = 230°C$ during hydrogenation for a different time should not affect the PL emission intensity. So far, the effects of ion beam heating in nanowires are unknown, however it might impact the diffusion speed of H in the lattice, or reactivate H atoms trapped at defects or in N-H complexes.

Figure 5 shows PL spectra of 3 different points on nanowires that were hydrogenated with different ion beam energies, where energy of the impinging protons is varied for 80, 100, and 120 eV while keeping a constant H dose of $d_H = 0.8H_0$. This dose is low enough that the previously meantioned V_{Ga}-H emission band does not affect the discussion. (a-c) show spectra taken on nanowires with N concentrations of 0.6% and (d-e) with N concentrations of 1.6% respectively. The nanowires hydrogenated with beam energies of 80 eV show a less complete disappearance of the low energy bandgap emission of GaAsN, indicating a less complete passivation of the N atoms for both samples, while no significant differences are observed in a qualitative assessment of the PL spectra after 100 eV and the 120 eV treatments.

Figure 6 shows the quantitative analysis of the influence of different beam energies on the PL emission and is calculated as the average of 4 to 12 measurements at different points on several NWs. The error bars for the FWHM show the standard deviation of measurements on different nanowires, the error bars for changes in intensity reflect possible variations due to the alignment of the setup between measurements before and after hydrogenation. (a) shows the ratio of the maximum intensity of the emission bands above and below 1.39 eV for the 0.6% N (top) and 1.6% N (bottom) samples. This is a measure of how completely the hydrogenation has passivated all N atoms in the GaAsN shell and how many defect states emit at low energy. For both samples, we observe the highest ratio at a beam energy of 100 eV. The FWHM of the PL emission band at RT is shown in (b). We ob-

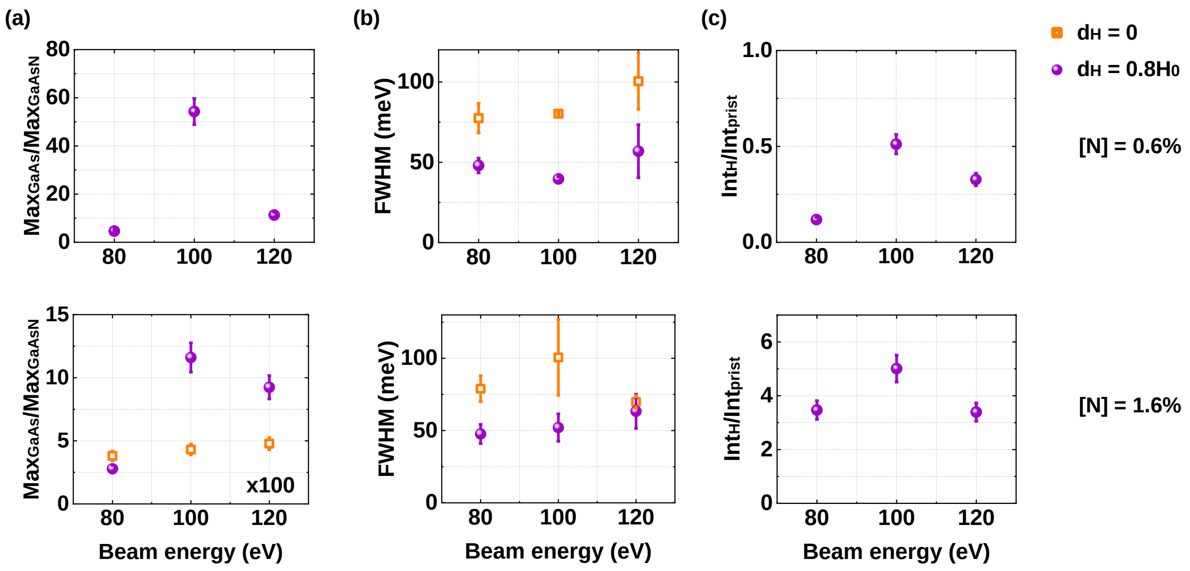

Figure 6: Quantitative analysis of the RT PL spectra from nanowires hydrogenated with different beam energies for nanowires with 0.6% N in the top row and 1.6% N in the bottom row. Some spectra are shown in Figure 5. (a) shows the ratio between the maximum RT PL intensity of the emission at the energy of the GaAs bandgap divided by the maximum intensity of the RT PL emission at the energy of the respective GaAsN bandgap. (b) shows the FWHM of the principal RT PL emission band before and after hydrogenation and (c) shows the ratio of the RT PL intensities integrated over the whole energy range before and after hydrogenation.

serve a small but consistent increase in FWHM with higher beam energy for both samples, independent of the differences shown by the untreated nanowires. To understand the effect of ion beam energy on the formation of non-radiative defects, we calculate the ratio of the integrated PL intensities of the hydrogenated and untreated nanowires and compare it for different beam energies, as shown in (c). Again, the highest value is for a beam energy of 100 eV. To summarize, it can be said that beam energies of more than 100 eV are advantageous for good spreading and distribution of the H atoms in the lattice. The use of energies above 100 eV leads to slightly more defects even if the hydrogenation time is shortened, but depending on the application, e.g. in the engineering of local quantum structures, these are not so crucial for good performance if they are not in the immediate vicinity of the structure. Therefore, a faster hydrogenation process and a shorter H-diffusion time at a higher beam energy, which enables the creation of sharper structures, could outweigh the disadvantages of slightly more non-radiative defects in the high bandgap material.

3.3 Hydrogenation at the slightly higher temperature of $T_H = 290°C$

Figure 7 investigates the impact of different substrate temperatures during hydrogenation. (a) and (b) show the PL spectra at RT for points on nanowires hydrogenated at $T_H = 230°C$ and $T_H = 290°C$, respectively. The top row shows measurements on points of nanowires with 0.6%N and the bottom row with 1.6%N. The nanowires were measured in the same point before and after hydrogenation. Both hydrogenation temperatures allow passivation of N atoms and shift the low energy GaAsN emission band to high energy GaAs emission. To understand the influence of the hydrogenation temperature on the FWHM and PL intensity, their averages are shown in (c) and (d) for both samples. The values of the FWHM of the untreated nanowires are also shown for comparing differences to the variations among nanowires. The FWHM of the peaks before and after hydrogenation is increasing a little for higher hydrogenation temperature in both samples. Nanowires from both samples show a slightly higher increase in PL intensity when hydrogenated at lower temperature of $T_H = 230°C$. This might be related to some non-radiative defects, which are passivated by a low temperature hydrogenation but are unpassivated at high temperatures.

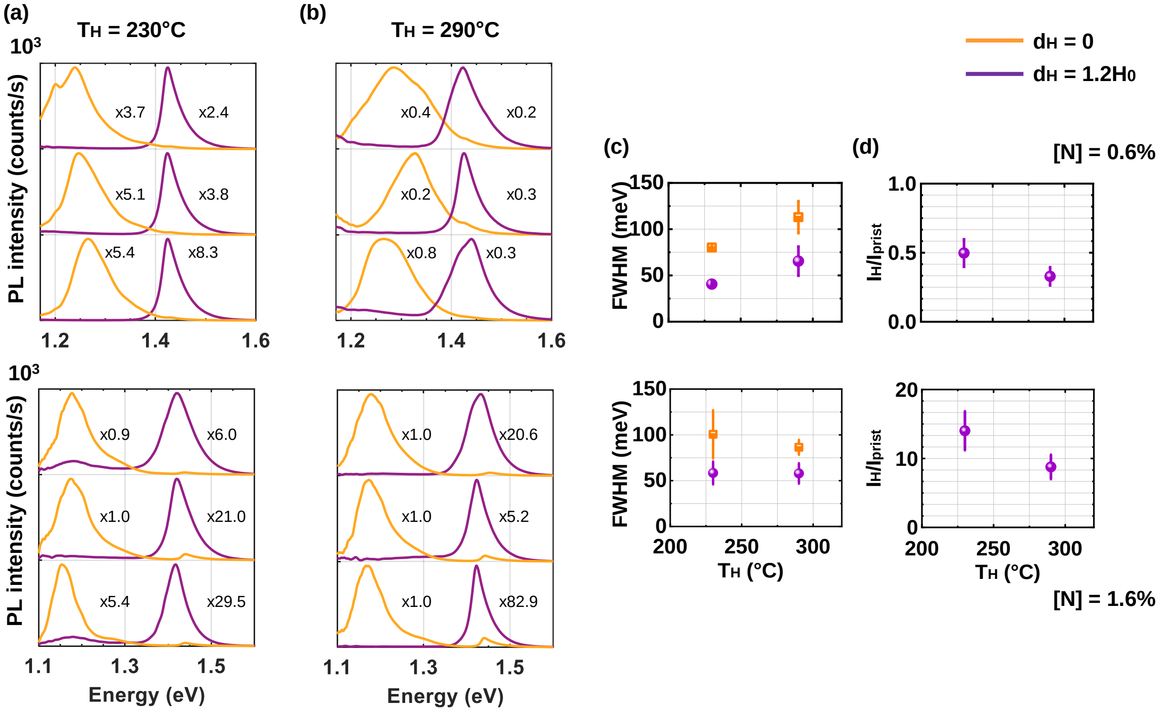

Figure 7: Effects of hydrogenation at different substrate temperature for a constant H-dose of $d_h = 1.2H_0$ for nanowires with 0.6% N in the top row and 1.6% N in the bottom row. (a) shows the RT PL spectra before (orange) and after (purple) hydrogenation at $T_H = 230$°C and (b) at $T_H = 290$°C. The spectra were divided by the maximum PL intensity as indicated by the factors. (c) shows the FWHM and (d) the intensity gain of the RT PL emission upon hydrogenation at different temperatures for the nanowires with 0.6% and 1.6% N.

4 Scan along nanowires with 0, 0.6 and 1.6 % N in the GaAsN shell

The main article discusses local variations in a μ-PL scan along the axis of a nanowire with 1.6% N before and after hydrogenation, and here in **Figure 8** we show the normalized spectra giving rise to the contour plots in the main text. To obtain the energy plotted in Fig. 3 (e), we selected the maximum value in the area of interest and fitted a Gaussian in that area to minimize noise and possible peak shifts due to a non-Gaussian shape. For the FWHM plotted in Fig. 3 (f), we took the difference between the two energy values at the half-height of the peak for both the GaAs and GaAsN emission bands.
Below, scans along nanowires with 0.6% N and along a GaAs reference nanowire are discussed, to support the trends discussed in the main for the 1.6 %N sample.

0.6% N in the GaAsN shell: **Figure 9** (a) shows the μ-PL scan at RT along the axis of a untreated nanowire with 0.6% N; (b) shows the μ-PL scan at RT along the same nanowire after hydrogenation with a H-dose of $d_H = 0.8H_0$. All spectra in the map have been normalized to 1 to allow observing variations in peak energy and FWHM. The PL intensities can be observed in (c), where the maximum intensity of each RT PL spectrum of the scans is given in counts/s. The PL intensity along the entire nanowire is reduced after hydrogenation, as observed for single point measurements on nanowires with 0.6% N shown in Figure 4 (c), Figure 6(c) and Figure 7(d). The bandgap energy and the FWHM of the bandgap emission as a function of the position on the nanowire are shown in (d) and (e), respectively. The bandgap energy is uniformly shifted to higher energy along the entire nanowire due to hydrogenation. The FWHM of the emission peak varies along the nanowire following the same pattern before and after hydrogenation, meaning that these variations are not due to differences in N-concentrations or impacted by the H incorporation, but caused by other crystal variations. As discussed in the main article for the sample with 1.6% N and in line with the results shown in Figure 3, H introduces a line narrowing along the entire length of the nanowire.

GaAs reference nanowires (0% N in the GaAsN-shell): **Figure 10** (a) shows the μ-PL scan along the axis of a untreated GaAs reference nanowire with 0% N. (b) shows the μ-PL scan along the same nanowire after hydro-

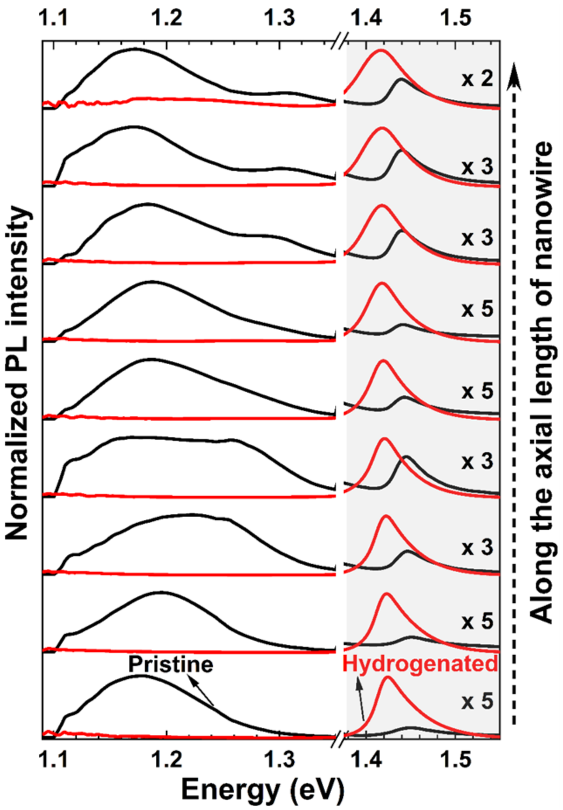

Figure 8: Stacked μ-PL plots resulting in the scan shown in Figure 3 of the main article for a nanowire with 1.6% N before (black spectra) and after (red spectra) hydrogenation. In the pristine spectra, the GaAs luminescence region (light grey-shadowed region) was multiplied by the given factor for each spectrum, depending on its relative intensity with respect to the GaAsN luminescence.

genation with a H-dose of $d_H = 0.8H_0$. All spectra in the map have been normalized to 1. The normalization factors are shown in (c) as the maximum intensity of the locally measured bandgap emission of the nanowire in counts/s. Due to hydrogenation, the points along the nanowire gain in intensity by approximately a factor 5, in agreement with what shown in Figure 4 (e). The bandgap energy along the nanowire, plotted in (d), is constant and approximately equal to 1.423 eV after hydrogenation, while it shows some variation before hydrogenation between 1.423 and 1.43 eV. The FWHM of the bandgap emission in (e) shows a small increase due to hydrogenation in line with what is shown in Figure 3. As these phenomena are observed only in certain points and disappear after removing H from the lattice through thermal annealing as shown later on, they are related to the presence of H in the lattice and might be due local electric fields created through H piling around a defected core-shell interface as observed at interfaces in planar structures[5].

5 Laser Annealing - single spectra

Figure 11 shows the single spectra of the maps shown in Figure 5 of the main paper. (a) shows the μ-PL spectra of the untreated nanowire, (b) after hydrogenation and (c) after being laser annealed at a coordinate of approximately 2.5 μm of the scan. The pristine nanowire shows the usual high intensity-low energy GaAsN band and the low intensity-high energy GaAs band. Hydrogenation leads to GaAsN passivation, resulting in the increase of the high energy band. The low energy band is still partially visible because of the non complete passivation due to the intermediate H-dose of $0.8H_0$, which is why a dose of $0.4H_0$ was added before laser annealing. After laser annealing, several H-N complexes are broken leading to unpassivated N atoms shifting the bandgap to lower energies. Indeed, the bandgap emission of GaAsN is restored close to the point of the laser annealing, while the bandgap emission of the GaAs-like peak remains intact on the other end. The bandgap of the annealed GaAsN-H has a slightly higher energy than the GaAsN band of the untreated nanowire, which indicates that a certain amount of H atoms are still being bound in the GaAsN lattice. The area affected by the laser annealing is quite large due to the extension of the laser beam of approximately 700 nm and due to heat conduction within

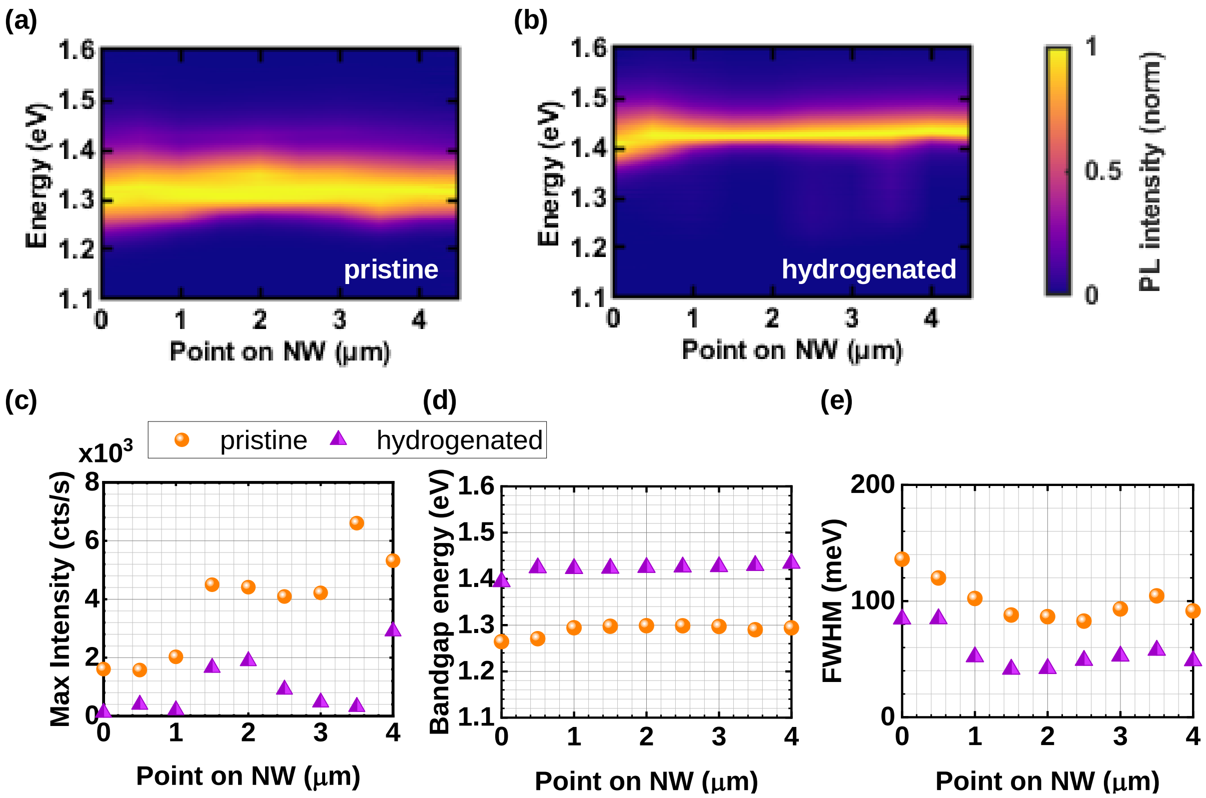

Figure 9: µ-PL scan along a nanowire with 0.6% N in the GaAsN shell, before (a) and after hydrogenation (b). Each spectrum is normalized to 1. The maximum of the peak intensity in each point along the nanowire axis (the normalization factor) is shown in (c). The bandgap energy and the FWHM of the bandgap emission along the nanowire are shown in (d) and (e). The hydrogenation dose is $d_H = 0.8H_0$.

the nanowire being larger than the heat conduction from the nanowire to the substrate. The procedure can be optimized using a dielectric tip to focus the laser beam on a smaller spot, and for better heat evacuation from the nanowire to the Si substrate.

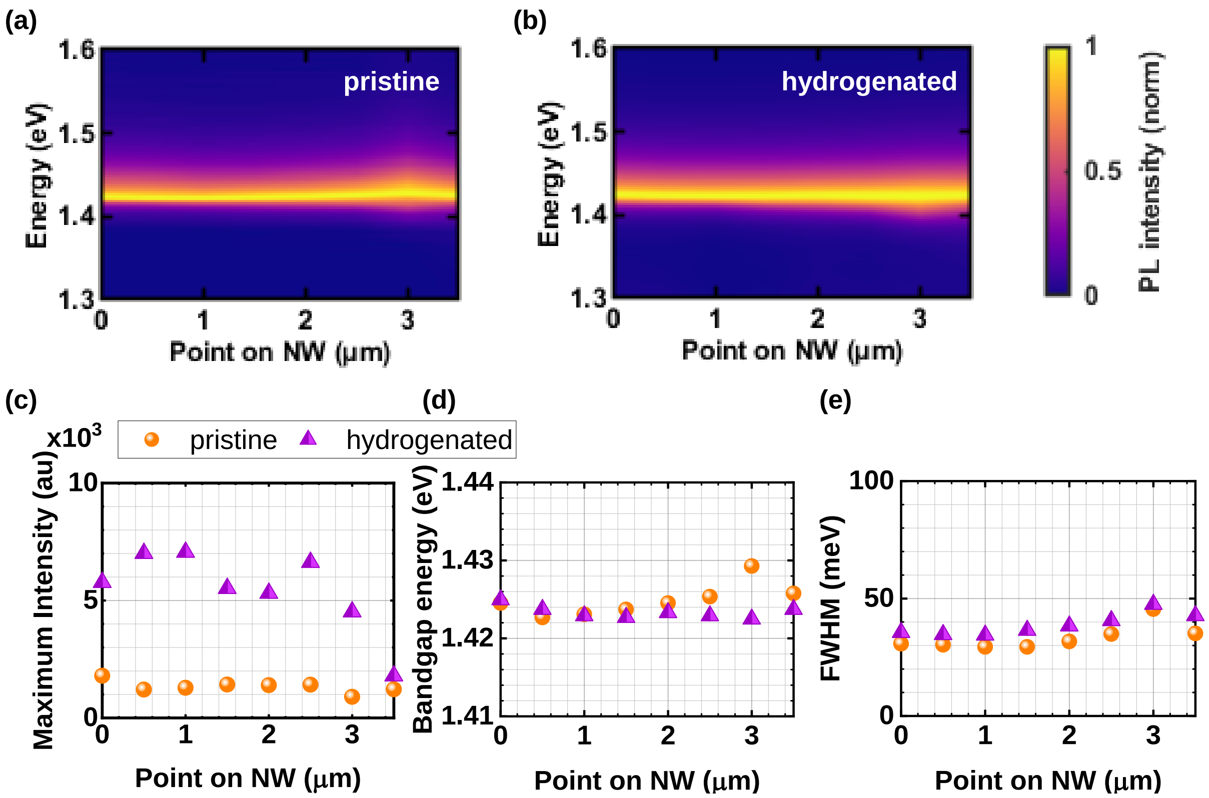

Figure 10: µ-PL scan at RT along a GaAs reference nanowire, measured before (a) and after (b) hydrogenation with a dose of $d_H = 0.8H_0$. Each spectrum is normalized to 1. The maximum of the peak intensity in each point along the nanowire axis, which corresponds to the normalization factor, is shown in (c). The extracted bandgap energy and the FWHM of the bandgap emission along the nanowire are shown in (d) and (e) as a function of the position along the NW axis.

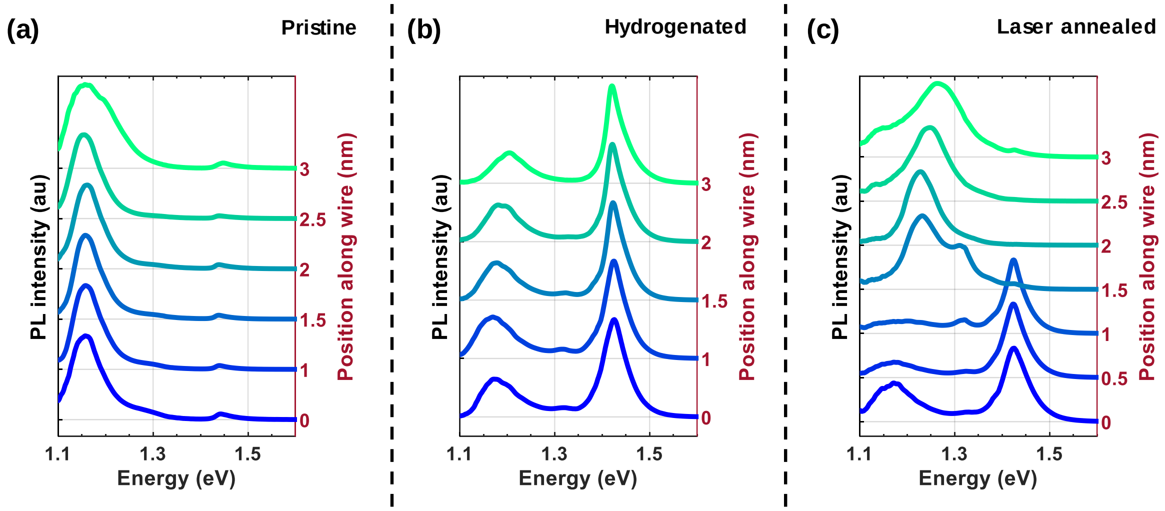

Figure 11: Stacked plots resulting in the scans shown in Figure 5 of the article for a nanowire with 1.6% N before hydrogenation (a), after hydrogenation (b) with 0.8 H_0, and after being laser annealed in the right part of the nanowire (c). The spectra are taken at RT, with a laser power $P = 10\mu W$ and measured with the CCD detector, which is responsible for the spectral cut on the low energy side. The power used for the laser annealing was up to $P = 1000\mu W$ at cryogenic temperatures while monitoring the PL emission.

References

[1] J. Wu, W. Shan, W. Walukiewicz, *Semiconductor Science and Technology* **2002**, *17*, 8 860.

[2] I. Vurgaftman, J. R. Meyer, L. R. Ram-Mohan, *Journal of Applied Physics* **2001**, *89*, 11 5815.

[3] I. Vurgaftman, J. R. Meyer, *Journal of Applied Physics* **2003**, *94*, 6 3675.

[4] W. Shan, W. Walukiewicz, J. W. Ager, E. E. Haller, J. F. Geisz, D. J. Friedman, J. M. Olson, S. R. Kurtz, *Phys. Rev. Lett.* **1999**, *82*, 6 1221.

[5] M. Capizzi, V. Emiliani, A. Frova, F. Sarto, R. N. Sacks, *Phys. Rev. B* **1993**, *47*, 19 12563.
